# Bile Salts in Chiral Micellar Electrokinetic Chromatography: 2000–2020

**DOI:** 10.3390/molecules26185531

**Published:** 2021-09-12

**Authors:** Raymond B. Yu, Joselito P. Quirino

**Affiliations:** Australian Centre for Research on Separation Science (ACROSS), School of Natural Sciences-Chemistry, University of Tasmania, Hobart 7001, Australia; raymond.yu@utas.edu.au

**Keywords:** bile salts, capillary electrophoresis, chiral selector, chiral separation, electrokinetic chromatography, surfactants

## Abstract

Bile salts are naturally occurring chiral surfactants that are able to solubilize hydrophobic compounds. Because of this ability, bile salts were exploited as chiral selectors added to the background solution (BGS) in the chiral micellar electrokinetic chromatography (MEKC) of various small molecules. In this review, we aimed to examine the developments in research on chiral MEKC using bile salts as chiral selectors over the past 20 years. The review begins with a discussion of the aggregation of bile salts in chiral recognition and separation, followed by the use of single bile salts and bile salts with other chiral selectors (i.e., cyclodextrins, proteins and single-stranded DNA aptamers). Advanced techniques such as partial-filling MEKC, stacking and single-drop microextraction were considered. Potential applications to real samples, including enantiomeric impurity analysis, were also discussed.

## 1. Introduction

The importance of chiral separation as an analytical technique cannot be understated. The different biological activities or potencies of chiral chemicals such as pharmaceuticals and agrochemicals are well documented and thus give impetus to chiral separation. In response to this, various analytical separation techniques have been developed in the fields of liquid chromatography, gas chromatography and capillary electrophoresis (CE). In chiral CE, additives called chiral selectors (CS) are usually added into the separation media or background solution (BGS). CSs act as the pseudophase, which interacts selectively with the enantiomeric analytes. The selective interaction causes chiral separation. Various CSs have been developed and/or utilized in chiral CE separations, such as antibiotics, ionic liquids, ligand exchangers, oligonucleotide aptamers, sugars (e.g., cyclodextrins (CDs)) and surfactants [1,2,3,4,5,6,7,8].

Surfactants at concentrations above the critical micelle concentration (CMC) form micelles in solution. These micelles were used as a chromatographic pseudophase in CE in the technique called micellar electrokinetic chromatography (MEKC) introduced by Terabe and co-workers in 1984 [9]. In 1989, Terabe’s group also introduced bile salts as chiral surfactant additives in MEKC for the separation of derivatized amino acids [10] and chiral drugs [11]. Bile salts are biosurfactants derived from the hepatic biotransformation of cholesterol. These compounds act to emulsify and solubilize fatty acids, monoglycerides, cholesterol and fat-soluble vitamins in human and mammalian digestive systems [12,13]. As such, they have also been shown to solubilize various lipophilic molecules, such as drugs [14,15], vesicles [13] and model compounds [16]. Following the use of bile salts as CS in MEKC, other naturally derived chiral surfactants such digitonin and saponins and synthetic surfactants such as amino-acid-based surfactants, glycosidic surfactants and molecular micelles have been utilized for the chiral MEKC of small molecules.

**Figure 1:**
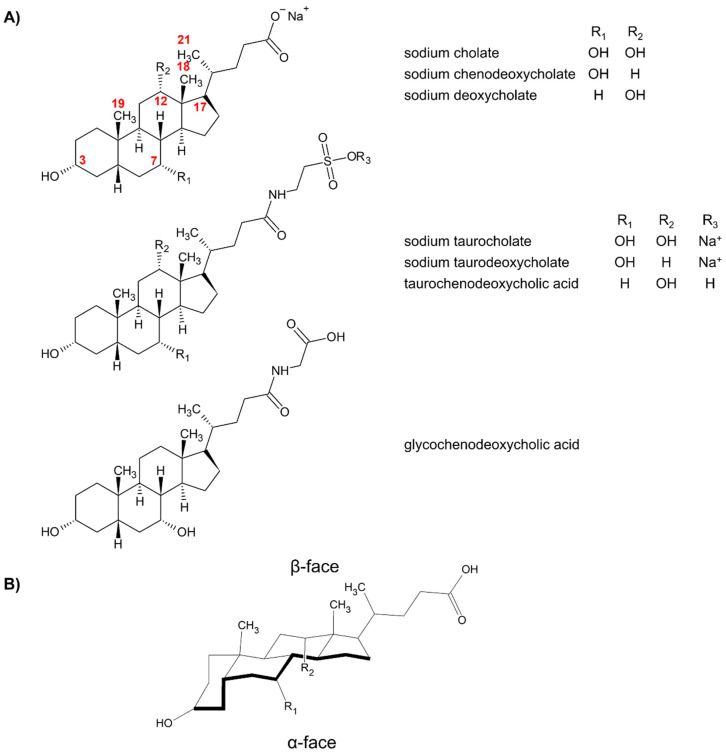
(**A**) Structures of bile acids commonly utilized in MEKC from 2000 to 2020. The locants of important positions in sodium cholate or sodium deoxycholate have been indicated. (**B**) Location of the α- and β-faces of bile salts using cholic acid as example. Figure 1B was reprinted with permission from [17], © 2021 American Chemical Society.

Figure 1A shows the bile salts used in MEKC from 2000 to 2020. In general, the bile salts are large, rigid and planar molecules containing a saturated steroidal ring structure. Different bile acids are differentiated by the number and stereochemistry of hydroxyl groups in the ring, and a carbon side chain attached to C-17. The steroidal ring contains between one and three hydroxyl groups located at carbons 3, 7 or 12. These hydroxyl groups are mostly oriented to the plane lying beneath the equator. This plane is called the α-face (see Figure 1B). The α-face is the hydrophilic surface of bile salts. The opposite face is the β-face. Methyl groups are oriented towards the β-face and thus constitute the hydrophobic surface of the bile salts. On the other hand, the carbon side chain contains the ionic head. Primarily, the ionic head gives the bile salts charge. The ionic head can be unconjugated like sodium cholate or can be conjugated with taurine like sodium taurocholate or with glycine like glycochenodeoxycholic acid.

Several reviews have been dedicated to the use of various chiral surfactants such as molecular micelles [18,19,20,21,22] and glycosidic surfactants [23]. However, a Scopus search revealed that no reviews were made for bile salts since the review by Otsuka and Terabe two decades ago [24]. Therefore, in this review, we will discuss the developments in research on bile salt as chiral surfactants in MEKC from 2000. Four different databases (Pubmed, SciFinder, Scopus, and Web of Science) were used to search for articles. The search terms used were (“bile salts” OR “micelles”) AND (“chiral capillary electrophoresis” OR “chiral micellar electrokinetic chromatography”) (from the year 2000). Altogether, the search turned out an average of 500 papers per search engine, including overlaps. Only papers that successfully utilized bile salts as sole or ancillary CS in MEKC were selected and included in this review. All in all, 44 papers were found in this important topic. These papers will be discussed in this review.

## 2. Aggregation Behavior and Mechanism of Chiral Recognition of Bile Salts

Bile salts aggregate into micelles upon reaching the CMC. Table 1 lists the CMCs of various bile salts used in MEKC from 2000 to 2020. Unlike the more common long alkyl chain surfactants (e.g., cetyltrimethylammonium bromide or sodium dodecyl sulfate (SDS)) whose amphipathicity arises from its hydrophilic head and hydrophobic tail, the amphipathicity of bile salts arises from its hydrophilic and hydrophobic faces. As a result, bile salt micelles have a facial structure [25]. Micellar structures such as primary and secondary structures as well as disc-like and helical micelles have been proposed, although it is the primary and secondary structures that have been demonstrated in several experiments [25].

The aggregation behavior of bile salts was first described by Small and co-workers [31]. Bile salts were initially thought to aggregate in a stepwise manner into bigger structures. These were called the primary and the secondary micelles. This was confirmed by Rovnyak and co-workers in a series of four papers using data gathered from MEKC and proton nuclear magnetic resonance spectroscopy (^1^H NMR) [16,17,32,33]. They used *R,S*-binaphthyl-1,1′-diylhydrogenphosphate as an analyte. For sodium cholate, they noticed the absence of chiral resolution of the analyte below a micelle concentration of ~14 mM. This concentration was observed to be the CMC of the primary micelle in aqueous solution. Interestingly, independent research from Hu and co-workers using palonosetron hydrochloride as an analyte probe arrived at 10 mM as the primary CMC of sodium cholate [34]. However, there was an observable degradation in chiral resolution in high sodium cholate concentrations (≥30 mM). For sodium deoxycholate, chiral resolution was observed as early as 3 mM, while degradation in chiral resolution was observed at 20 mM.

**Figure 2:**
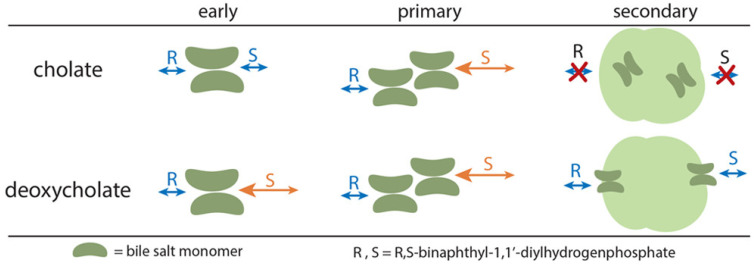
Schematic diagram of the aggregation behavior of bile salts using *R,S*-binaphthyl-1,1′-diylhydrogenphosphate as probe. Bile salts aggregate stepwise into pre-micellar aggregates, primary and secondary micelles with increasing bile salt concentration. The *S*-isomer is preferentially bound in these bile micelles and enter through the 12α-OH edge. Secondary micelles of sodium cholate and deoxycholate are non-enantioselective yet have different binding surface structures. Reprinted with permission from [17], © 2021 American Chemical Society.

To further understand the aggregation behavior of sodium cholate and sodium deoxycholate, the same authors used ^1^H NMR. The ^1^H NMR data suggested that bile salts dimerize, with the hydrophilic α-face exposed to the surface and the hydrophobic β-faces of the bile salts facing each other (see Figure 1B for the location of the faces). Below the primary CMC, a pre-micellar aggregate was formed. The pre-micellar aggregate of sodium cholate was formed around 7 mM and was not enantioselective. On the other hand, the pre-micellar aggregate of sodium deoxycholate was formed at 3 mM and is enantioselective. Increasing the concentration of sodium cholate to 14 mM and sodium deoxycholate to 7 mM, the formation of the primary micelles was observed. ^1^H NMR data confirm that the formation of primary micelles was required for chiral recognition by sodium cholate. Enantioselectivity arose from the preferential binding of a stereoisomer to a specific edge of the bile salt, with the *S*-isomer preferentially binding to the 12α-OH edge, and the *R*-isomer preferentially binding to the 7α-OH edge (see Figure 2).

At higher concentrations, the formation of much larger secondary micelles was confirmed. Unlike primary micelles, secondary micelles were not enantioselective. ^1^H NMR data showed that the binding pockets of sodium cholate secondary micelles were not available for binding. It was hypothesized that the hydrophilic α-face of sodium cholate secondary micelles was displayed on the surface, which blocks the analytes’ access to the binding site. Surprisingly, the binding pockets of sodium deoxycholate secondary micelles were conserved. It was proposed that both *R* and *S*-isomers have equal access to the binding site of sodium deoxycholate, which explained the degradation of chiral resolution. Although not confirmed for other bile salts, the development of chiral MEKC at high concentrations of bile salts can be avoided.

In addition, it was suggested that the 12α-OH moiety was needed for chiral recognition [16]. Sodium cholate, sodium deoxycholate and sodium chenodeoxycholate were tested for the chiral MEKC of *R,S*-binaphthyl-1,1′-diylhydrogenphosphate. Sodium chenodeoxycholate, which did not contain a 12α-OH moiety, was unable to affect chiral separation on the said analyte.

## 3. General Considerations on Chiral MEKC with Bile Salts

**Figure 3:**
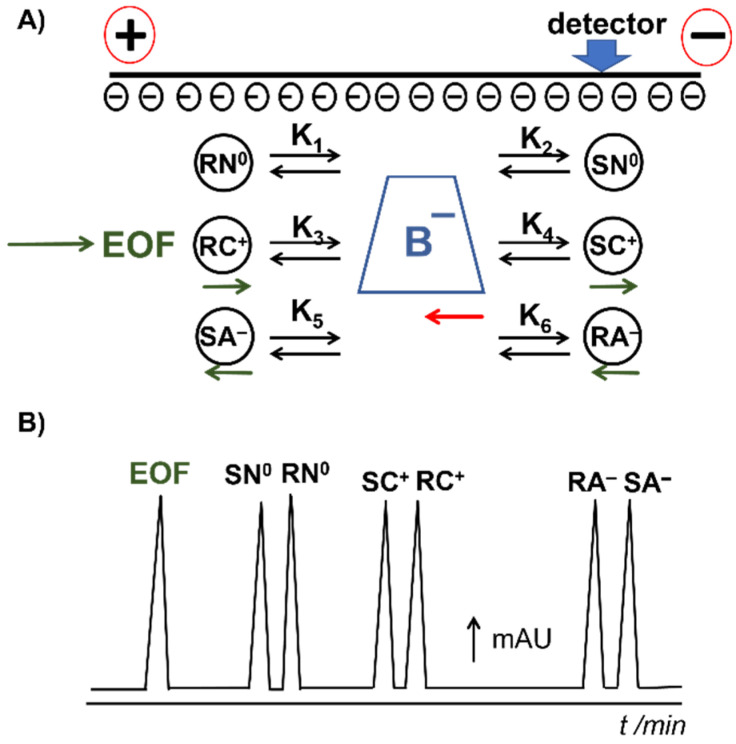
(**A**) Schematic on the chiral MEKC separation using bile salts of three hypothetical chiral species, neutral N^0^, cationic C^+^ and anionic A^-^, in their *R* and *S* enantiomers. (**B**) Resulting electrochromatogram of the chiral MEKC separation of the three pairs of analytes.

Bile salts are the most commercially available chiral surfactants. Bile salts are salts of weak acids with pKa ranges of 4.2–7.3 [35]. Therefore, they ionize and form aggregates in solution with pHs from neutral to alkaline. Figure 3 shows the general separation scheme when bile salts are used in chiral MEKC with neutral to alkaline buffers. Figure 3A shows the direction of the electroosmotic flow (EOF) and the electrophoretic mobility of neutral (N^0^), cationic (C^+^) and anionic (A^−^) charged analytes and the negatively charged bile acid (B^−^) at positive polarity separation. The detector is at the cathodic side. The strong EOF carried all analytes and bile salts to the detector. The analytes were detected after the EOF (see Figure 3B). The enantiomers of each analyte were separated by differential partitioning with the bile salt micelles. In the example in Figure 3A, the partition coefficient of RN^0^, K_1_ > K_2_, of RC^+^, K_3_ > K_4_, and of SA^-^, K_5_ > K_6_. Thus, in the theoretical electrochromatogram in Figure 3B, SN^0^, SC^+^ and RA^-^ were detected before RN^0^, RC^+^ and SA^-^, respectively.

## 4. Method Development

Table 1 summarizes the various MEKC methods using bile salts for various analytes, such as derivatized amino acids and dipeptides, model compounds, pharmaceuticals (palonosetron hydrochloride, metyrosine, sertraline, praziquantel), pesticides (*cis*-bifenthrin) and environmental pollutants (chiral polychlorinated biphenyls or PCBs). The bile salts were either solely CS or aided by another CS. The analytes, the bile salt used, other CS used (if any), MEKC conditions, summary of outcomes and the corresponding references were provided. The composition of the BGS (type and concentration of bile salt, type and concentration of additives (another CS, organic solvent, surfactant), buffer concentration and pH), sample loading (injection time and pressure/voltage and sample concentration), applied voltage and capillary temperature were optimized to develop a suitable method. The nature of CS is one of the most important considerations in developing an MEKC method. For this reason, we structured our discussion based on the CS. These are bile salts as sole CS and bile salts with another CS. Special techniques such as partial-filling MEKC, stacking and single-drop microextraction (SDME) techniques were also discussed.

### 4.1. Bile Salts as Sole CS

Sodium cholate was the most prominent bile salt reported during this review period (13 papers). Asztemborska and co-workers used sodium cholate in the separation of the chiral flavanone glycosides naringin and neohesperidin [36]. In the study by Tian and co-workers, the chiral separation of palonosetron hydrochloride enantiomers was affected by sodium cholate [37]. Meanwhile, Hu and co-workers investigated the separation mechanism of the same drug at varying sodium cholate concentrations [34], while Guo and co-workers introduced a thermodynamic model to elucidate the migration order of palonosetron hydrochloride enantiomers [38]. In another study, Trapp used sodium cholate with the probe ferroin (tris(1,10-phenanthroline)-Fe(II)) complex to investigate the effect of temperature and Joule heating in MEKC [39]. Sodium cholate was also used to investigate the enantiomerization barrier of the drugs lorazepam, oxazepam and temazepam [40] and the phytochemicals naringin and neohesperidin [41].

**Figure 4:**
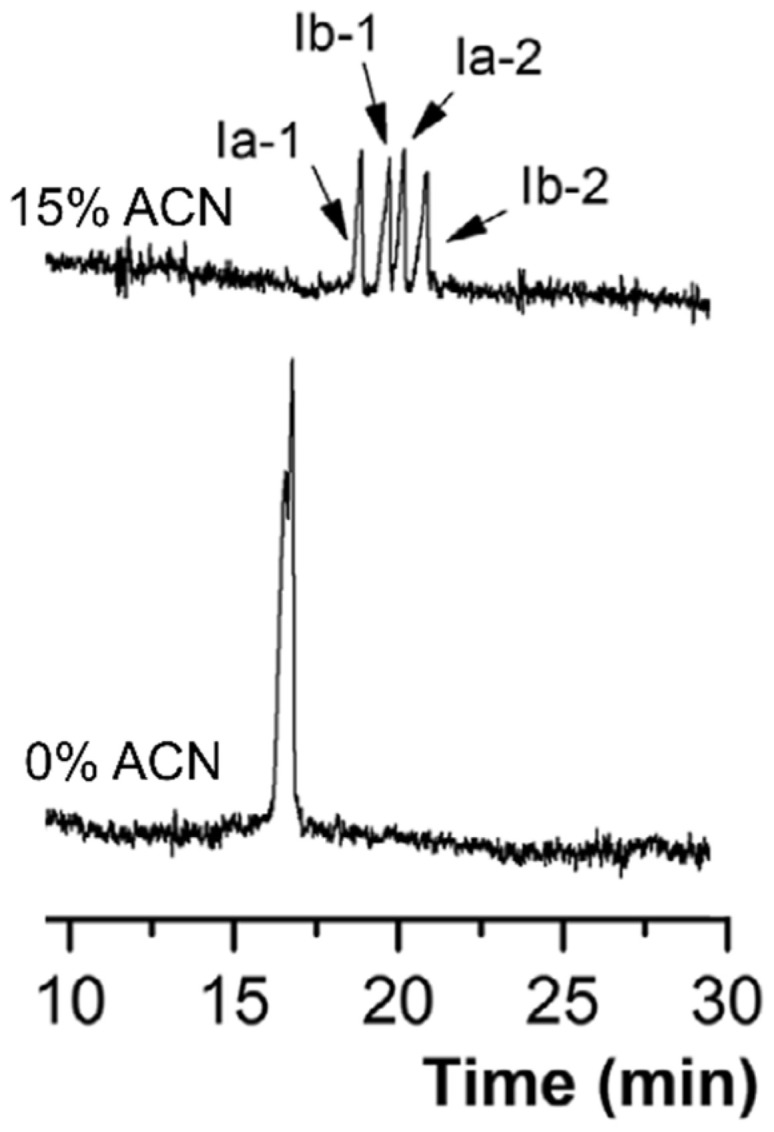
Electrochromatogram of the chiral separation four verteporfin enantiomers. Separation conditions: BGS: 25 mM sodium cholate in 120 mM Tris (pH 7.5) with or without 15% ACN. Sample injection: 0.5 psi for 5 s. Voltage: 15 kV. Detection: 428 nm. Capillary dimension: 50 cm (40 cm effective length) × 75 µm. Modified with permission from the authors [42], © 2021 John Wiley and Sons.

**Figure 5:**
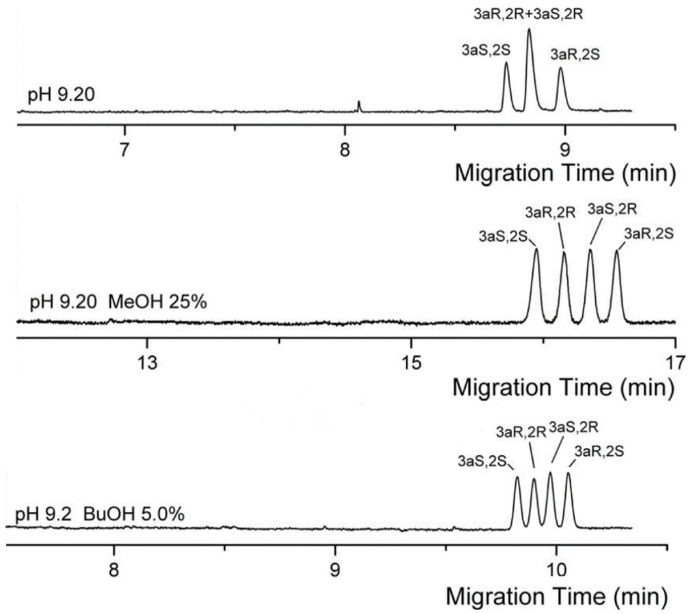
Electrochromatogram of the chiral separation four palonosetron hydrochloride enantiomers. Separation conditions: BGS: 70 mM sodium cholate in 30 mM sodium tetraborate (pH 9.2) with or without 25% methanol or 5% *n*-butanol. Sample injection: 10 kPa for 1 s. Voltage: 25 kV. Capillary temperature: 25 °C. Detection: 254 nm. Capillary dimension: 60 cm (50 cm effective length) × 50 µm. Modified with permission from the authors [44], © 2021 John Wiley and Sons.

Organic solvents (e.g., acetonitrile or methanol), surfactants (e.g., sodium dodecyl sulfate (SDS)) and co-surfactants (e.g., *n*-butanol) were added to improve the chiral separation of analytes. Figure 4 shows the effect of acetonitrile in the separation of verteporfin enantiomers conducted in the study by Xu and co-workers [42]. Baseline separation of the four enantiomers were observed with the aid of 15% (*v/v*) acetonitrile in the BGS containing 25 mM sodium cholate. This was not observed with sodium cholate alone (see Figure 4). Similarly, in the study of Peng and co-workers, the addition of 10–15% (*v/v*) acetonitrile in a BGS containing 25 mM sodium cholate improved the separation of three enantiomeric pairs of benzoporphyrin derivatives [43]. On the other hand, Hu and co-workers implemented various solvents (see Table 2 for complete list) [44,45] and SDS [46] to improve the separation of four palonosetron hydrochloride enantiomers. In the absence of organic solvent or SDS, the four enantiomers were not baseline resolved. Changes in the effective electrophoretic mobility due to organic solvent contributed to the improved separation. Figure 5 demonstrates the separation of four enantiomers in the presence of methanol or butanol. On one hand, the organic solvent or SDS improved the separation of the achiral diastereomeric pair (3aR,2R) and (3aS,2R). Interestingly, in the study of Huang and co-workers, the chiral MEKC of pyraclofos using high concentrations of sodium cholate with SDS was implemented using a non-aqueous media [47]. The use of non-aqueous MEKC might have been driven by the hydrophobicity of this analyte.

The other bile salts were sodium taurocholate, sodium taurochenodeoxycholate and sodium glychochenodeoxycholate. Sodium taurocholate was used for the enantiomers of metyrosine [48] and arotinolol [49], while sodium taurochenodeoxycholate and sodium glychochenodeoxycholate were used for Fe(II) complexes [50,51]. However, the MEKC separations of metyrosine and arotinolol were implemented under acidic pH, which makes the bile salts neutral and will less likely form micelles in solution.

### 4.2. Bile Salts with Another CS

In this section, we describe the use of bile salts in conjunction with another chiral additive. Mostly CDs were the other chiral additive used, but other CSs were also explored, such as human serum albumin and a 34-mer single stranded DNA (ssDNA) (see Section 4.3). CDs are cyclic oligosaccharides made up of 6–8 repeating glucose units linked together by α(1→4) glycosidic linkages. These compounds have a toroidal-shaped structure with a hydrophobic interior and a hydrophilic exterior. CDs can form inclusion complexes with small molecules. This complexation process is stereospecific when the analyte is chiral. One enantiomer will have a higher affinity for the CD than the other and forms the basis of chiral recognition. The studies covered in this review used native (β- and γ-CD) and derivatized (2-hydroxypropyl-β-CD, sulfated β-CD, heptakis (2,3,6-tri-*O*-methyl)-β-CD) CDs together with bile salt.

**Figure 6:**
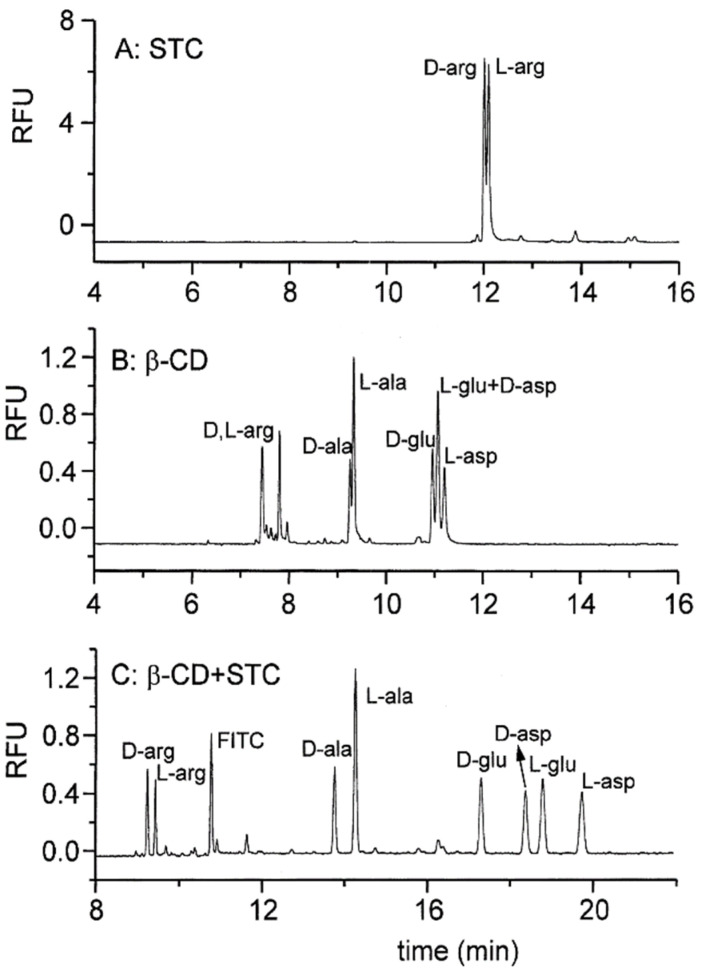
Electrochromatogram of the chiral separation of FITC-derivatized DL-amino acids. Separation conditions: separation media: 80 mM borate buffer (pH 9.3) with 30 mM sodium taurocholate (**A**), 30 mM β-CD (**B**), and 12 mM sodium taurocholate with 8 mM β-CD (**C**). Sample injection: 0.5 psi for 1 s. Voltage: 20 kV. Capillary temperature: 20 °C. Detection: 488 nm (λ_ex_) and 520 nm (λ_em_). Capillary dimension: 57 cm (50 cm effective length) × 50 µm. Reprinted with permission from the authors [58], © 2021 Elsevier.

In many cases, neither bile salt nor another CS could affect the chiral separation of mixtures. This is shown in Figure 6A,B using sodium taurocholate and β-CD as the sole CS in the MEKC of arginine (arg), respectively. The combination of sodium taurocholate and β-CD resulted in complete chiral separation (see Figure 6C) [58]. A similar result could be achieved using an achiral surfactant such as SDS with β-CD, as shown in the study by Giuffrida and co-workers [74]. However, better enantioseparations were observed with sodium taurocholate as a surfactant. Similarly, the chiral separation of 11 *o*-phthaldialdehyde- [59] and 19 9-fluorenylmethylchloroformate (FITC)- [71] derivatized amino acids was improved using sodium taurocholate and β-CD in the BGS. Zhao and co-workers developed a method for the determination of naphthalene-2,3-dicarboxaldehyde-derivatized D/L-serine (CBI-D/L-ser) using sodium deoxycholate and β-CD [64]. CBI-D/L-ser were only enantioseparated with both sodium deoxycholate and β-CD in the BGS. Meanwhile, Cheng and co-workers [65] initially used sodium deoxycholate to separate (*RS*)-1-phenyltetrahydrogen isoquinoline without success. With the addition of β-CD, chiral separation was affected. However, we found that the peak areas of the two enantiomers were not equal, despite the sample being a racemic mixture. Lastly, microfluidic MEKC enantioseparations of selected amino acids were implemented using sodium taurocholate and β-CD [60,61].

Two studies used γ-CD with bile salts as CS. Creamer and co-workers utilized sodium taurocholate and γ-CD for the analysis of seven 5-carboxyfluorescein succinimidyl ester-derivatized neutral amino acids [63]. Meanwhile, Huang and co-workers used sodium cholate with γ-CD for the chiral MEKC separation of the organophosphorus pesticides profenofos, prothiofos and sulprofos [47]. It is of interest to note that Huang’s group used an aqueous-organic media to implement their method. This was perhaps to increase the solubility of the hydrophobic organophosphorus compounds.

Other researchers used derivatized CDs as ancillary CS. Chen and co-workers [66] and Lucangioli and co-workers [54] independently developed two different methods for the separation of *cis-trans* isomers of sertraline using sodium deoxycholate and hydroxypropyl-β-CD and sodium cholate and hydroxypropyl-β-CD with sulfated β-CD, respectively. Garcia and co-workers used sodium deoxycholate with acetyl-β-CD for the chiral MEKC of bioallentrhin [67]. In the work of Chen and co-workers [68], the baseline enantioseparation of naphthalene-2,3-dicarboxaldehyde-tagged dipeptides alanylglutamine (ala-gln), tyrosylleucine (tyr-leu) and tyrosylphenylalanine (tyr-phe) was achieved by the combination of sodium deoxycholate and 2-hydroxypropyl-β-CD. In the study by Polisei Jabor and Bonato [69], sodium deoxycholate was only able to separate achiral drug praziquantel and metabolite *trans*-4-hydroxypraziquantel. The addition of sulfated β-CD enabled the enantioseparation of praziquantel and *trans*-4-hydroxypraziquantel isomers. However, they did not show the ability of sulfated β-CD to perform enantioseparation on its own. Such a study could bolster the need for either CS to effectively separate its isomers. Pérez-Fernández and co-workers developed a method for the enantioseparation of *cis*-bifenthrin using sodium cholate and heptakis(2,3,6-tri-O-methyl)-β-CD [55]. They noted that due to solubility issues with the said analyte, CD alone cannot be evaluated since bile salt micelles were required to solubilize the very hydrophobic insecticide.

Two studies found the use of SDS to enhance the chiral recognition ability of a dual CS system (i.e., bile salt and CD). It was suggested that the additional interaction of analyte enantiomers with SDS micelles improved chiral separation. Pérez Méndez and co-workers explored the use of SDS, β-CD and taurodeoxycholic acid for the chiral MEKC of selenoamino acid derivatives [73]. Chiral separation was affected by the combined use of SDS, β-CD and tauroedoxycholic acid; something which was not observed using either SDS and β-CD, or taurodeoxycholic acid alone. Similarly, Crego and co-workers used SDS, sodium taurodeoxycholic acid and γ-CD for the chiral MEKC of chiral PCBs [72].

Notably, Wang and co-workers utilized sodium cholate with human serum albumin as CS for the separation of 5-(4,6-dichloro-*s*-triazin-2-ylamino) fluorescein (DTAF)-derivatized aspartic (DTAF-asp) and glutamic acid (DTAF-glu) [56]. The combination of sodium cholate and human serum albumin enabled the separation of DTAF-asp and DTAF-glu, which was not possible as individual CS.

An interesting study by Bielejewska and co-workers demonstrated the ability of bile salts to reverse the migration orders of the model molecule 1,1′-binaphthyl-2,2′-diyl hydrogenphosphate (BNP) in MEKC [75]. Using β-CD and hydroxypropyl β-CD, the *S*-BNP migrated first. With the addition of sodium cholate to β-CD, or sodium cholate sodium taurodeoxycholate to hydroxypropyl β-CD, *R*-BNP migrated first.

### 4.3. Advanced Techniques

The advanced techniques considered in this section are partial-filling MEKC, stacking or sample concentration in CE, and SDME. In typical MEKC, the entire capillary is filled with the BGS containing the CS. In partial-filling MEKC, only a fraction of the capillary is filled with the BGS containing the CS, and thus separation occurs in two ways. The first separation is by MEKC followed by capillary zone electrophoresis (CZE) after the analytes migrate out of the BGS containing the CS plug. CZE separation is due to differences in analyte electrophoretic mobilities.

**Figure 7:**
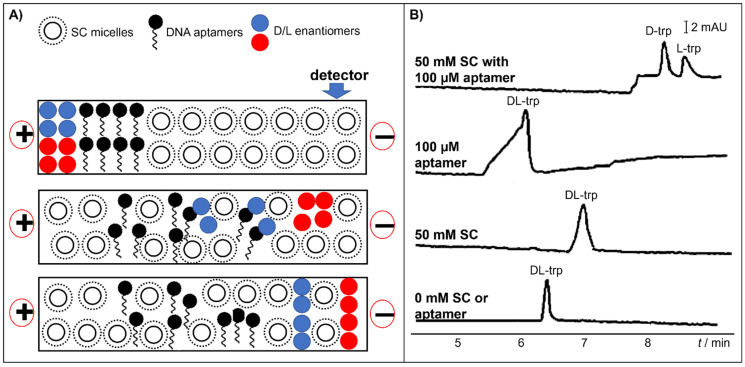
(**A**) Schematic diagram of the procedure of partial filling. (**B**) Electrochromatograms of DL-tryptophan under various CS concentration. BGS contains CS as indicated in each trace. The aptamer is a 34-mer ssDNA specific to DL-tryptophan. Separation conditions: buffer: 2.7 mM KCl, 1 mM MgCl_2_, 10 mM Na_2_HPO_4_, 10 mM NaCl, and 2 mM KH_2_PO_4_ (pH 7.4). Sample injection: 0.5 psi for 6 s. Applied voltage: 20 kV. Detection: 280 nm. Capillary dimension: 61 cm (51 cm) × 75 µm. Modified with permission from the authors [57], © 2021 John Wiley and Sons.

Huang and co-workers used sodium cholate in combination with DNA aptamer (i.e., a 34-mer ssDNA specific for DL-tryptophan) as CS for DL-tryptophan separation [57]. The DNA aptamer is a UV active molecule and thus will interfere with the detection of the amino acid. Thus, partial-filling MEKC was performed, and the principle for their separation is shown in Figure 7A. A capillary was first flushed with a BGS containing sodium cholate micelles. A plug of a solution containing the DNA aptamer was introduced into the capillary. Thus, only a certain length of the capillary was filled with the aptamer. Then, the sample was injected. Chiral separation arose due to the selective affinity of each analyte to the CS as the enantiomeric analytes travel through the capillary. When voltage was applied across the capillary, tryptophan migrated across the DNA aptamer plug. The L enantiomer (blue circle in Figure 7A) was more solubilized in the aptamer plug. Then, all tryptophan enantiomers migrated into the sodium cholate BGS zone (the D first then the L). As seen in Figure 7B, expectedly, there was no chiral separation in the absence of any of the CS. The chiral separation of tryptophan enantiomers was not achieved using the DNA aptamer or sodium cholate micelles alone. The combination of the aptamers and the sodium cholate micelles enabled the chiral separation of the said analyte.

Stacking in CE involves the long injection of a sample prepared in a special matrix. Upon the application of voltage, the analytes focus in a small zone inside the capillary and thus improve the poor detection sensitivity, especially with low analyte concentrations. Stacking techniques in MEKC include sweeping, field amplification and micelle-to-solvent stacking [76,77,78]. The extent of stacking is normally expressed by calculating the sensitivity enhancement factor (SEF). SEF is calculated by dividing the peak signal with stacking by the peak signal with short injection multiplied by the dilution factor, if any.

In the review period, three papers utilized stacking techniques to improve the detection of model analytes and amino acids. Choy and co-workers developed a method involving acetonitrile stacking coupled with MEKC using sodium cholate for the analysis of binaphthyl enantiomers and DL-tryptophan [52]. In this technique, the sample matrix contains acetonitrile and 1% sodium chloride. The technique enabled the preconcentration of low concentrations of analytes (0.2–20 µg/mL). However, the authors did not mention the SEF of their method, though we noted that the method could potentially offer 18-fold sensitivity enhancement. On the other hand, Lin and co-workers combined poly(ethylene oxide) (PEO)-mediated stacking and sweeping with MEKC using sodium taurocholate and β-CD, together with the derivatization of amino acids for the simultaneous analysis of amino acids [70]. In this technique, the sample was concentrated by sweeping by the anodically-moving bile salts. In addition, when the sample met the PEO zone, the analytes were believed to be additionally stacked due to the viscosity of the BGS. The authors were able to obtain notable SEFs between 106 and 219. Lastly, Xu and co-workers performed pressure-assisted electrokinetic injection (PAEKI) with MEKC using sodium cholate for the analysis of verteporfin [53]. Briefly, in PAEKI, the pressure applied at the capillary inlet counterbalances the EOF generated during EKI, stacking the analyte during the process. The authors noted a 116- and 39-fold improvement in LOD compared to typical hydrodynamic and electrokinetic injection.

SDME is an inline liquid–liquid extraction technique developed for CE. The sample in an acidic media is partitioned to a nanodrop containing the basic BGS and coated with an organic phase in the inlet end. Sample partitioning is driven by pH difference. Liang and co-workers coupled SDME with MEKC using sodium cholate of FITC-DL-alanine, FITC-DL-aspartate, FITC-DL-glutamate and FITC-DL-leucine [62]. SEFs as high as 3000–6800 were obtained, which was accomplished with a retrofitted microstirrer setup.

## 5. Applications to Real Sample Analyses

The potential application of MEKC with bile salts in real sample analyses was found in fourteen papers. Real sample matrices included beverage, biological samples (human and animal sera, neural samples and artificial urine), environmental samples, insecticides and pharmaceutical formulations. Amino acids derivatized and spiked in various beer samples were determined using MEKC with sodium taurodeoxycholate and β-CD, coupled with sweeping and PEO-mediated stacking [70]. Arotinolol [49] and dipeptides [68] spiked in human serum samples were determined using sodium taurocholate, and sodium deoxycholate with 2-hydroxypropyl-β-CD, respectively. Meanwhile, praziquantel and its metabolite were analyzed in actual serum samples from human volunteers who were administered with the said drug [69]. Benzoporphyrin derivatives extracted from bovine serum and liver microsomes were determined using sodium cholate [43]. D-serine from the neurons from the ganglia from *Aplysia* sp was determined using sodium deoxycholate and β-CD [64]. Verteporfin was determined in spiked artificial urine using sodium cholate [53]. Organophosphorus pesticides pyraclofos, profenofos, prothiofos and sulprofos were determined in spiked soil samples using sodium cholate either with SDS or γ-CD [47]. Neutral amino acid biosignatures from Lake Mono were determined using a combination of sodium taurocholate and γ-CD [63]. The commercial formulations of insecticides *cis*-bifenthrin [55] and bioallenthrin [67] were determined using sodium cholate with heptakis (2,3,6-tri-O-methyl)-β-CD and sodium deoxycholate with acetyl-β-CD, respectively. Moreover, bioallenthrin degradation studies were also performed. Lastly, the pharmaceutical agents palonosetron hydrochloride [46] and sertraline [66], and derivatized amino acids [56], were determined in their formulation using sodium cholate with SDS, sodium deoxycholate with hydroxypropyl-β-CD, and sodium cholate with human serum albumin, respectively.

Enantiomeric impurity analysis is an important application of chiral MEKC. During the review period, four papers implemented bile acids for the enantiomeric impurity analysis for sertraline [54,66], selenomethionine [73], and DL-alanine and DL-glutamine [62]. Two methods were developed for sertraline, namely, one using taurodexocycholic acid with β-CD [66] and sodium cholate with hydroxypropyl β-CD and sulfated β-CD [54]. Selenomethionine used taurodeoxycholic acid with β-CD and SDS [73]. FITC-derivatized DL-alanine and DL-glutamate used sodium taurocholate and β-CD [62].

## 6. Concluding Remarks

Bile salts were important CSs in the development of chiral MEKC methods for various analytes. In the 44 papers that were analyzed in this review, bile salts, either as sole CS or with another CS or another surfactant aided in the separation of various analytes, such as amino acids, pesticides, pharmaceutical drugs and phytochemicals. Bile salts were also implemented in real sample determinations in various matrices, such as food, biological and environmental samples, and pharmaceutical preparations. An advantage of bile salts is that they are the most commercially available chiral surfactants, while other chiral surfactants need to be synthesized. Despite the moderate usage of bile salts in chiral MEKC, these compounds remain relevant and important. It was clearly shown in this review that ineffective single CS systems were improved by the addition of bile salts. Enantioselectivity and solubilizing power of bile salt systems are attractive for the MEKC separation of enantiomers with low water solubility. Thus, the development of chiral MEKC with bile salts will continue in the future.

## Figures and Tables

**Table 1:** Bile acids commonly utilized in MEKC from 2000 to 2020, their CMC and references.

Bile Salt	CMC, mM ^a^	Ref.
Sodium cholate	13–14 (pH 12, 25 °C)	[17]
Sodium chenodeoxycholate	3 (pure water, 25 °C)	[26]
Sodium deoxycholate	5.4 (pure water, 25 °C)	[27]
Sodium taurocholate	8–12 (pure water, 25 °C)	[28]
Sodium taurodeoxycholate	3.5–6 (pure water, 25 °C)	[29]
Taurochenodeoxycholic acid	7 (pure water, 25 °C)	[29]
Glycochenodeoxycholic aicd	2 (pH 7.5, 25 °C)	[30]

^a^ CMC here refers to the primary CMC.

**Table 2:** Chiral surfactants used in MEKC, other CS involved, conditions used, summary of outcomes, analytes tested and their corresponding references.

Surfactant	Other Chiral Selector	MEKC Conditions	Summary of Outcomes	Analytes	Ref.
*As Sole Chiral Selector*					
Sodium Cholate	N/A	capillary dimension: 65 cm (59 cm effective length) × 75 μm BGS: 100 mM sodium cholate in 50 mM Tris and 50 mM borate buffer (pH 8.3) injection: 0.8 psi for 3 s capillary temperature: 20 °C separation voltage: 20 kV detection: 220 nm	-	naringenin, neohesperidin	[36]
		capillary dimension: 60 cm (52.5 cm effective length) × 50 μm BGS: 70 mM sodium cholate in 30 mM borate buffer (pH 9.4) with 20% (*v*/*v*) MeOH injection: 5 sec at a height of 10 cm (anodic) capillary temperature: 25 °C separation voltage: 20 kV detection: 254 nm	-	palonosetron hydrochloride	[37]
		capillary dimension: 80.5 cm (72 cm effective length) × 50 μm BGS: 46.5 mM sodium cholate in 50 mM sodium borate/sodium dihydrogen phosphate buffer (pH 8) injection: 50 mbar for 1 s capillary temperature: 10–30 °C separation voltage: 15–25 kV detection: 210 ± 5 nm	-	ferroin (tris(1,10-phenantroline)-iron(II) complex	[39]
		capillary dimension: 112 cm (95 cm effective length) × 50 μm BGS: 60 mM sodium cholate in 20 mM borate/phosphate buffer (pH 8) injection: 20 mbar for 2 s capillary temperature: 0–30 °C separation voltage: 25 kV detection: 230 nm	-	lorazepam, oxazepam, temazepam	[40]
		capillary dimension: 60 cm (51.5 cm effective length) × 50 μm BGS: 50 mg/mL sodium cholate in 25 mM borate/phosphate (pH 7 or 9) or 25 mM borate (pH 10) injection: 30 mbar for 3 s capillary temperature: 15 °C separation voltage: 20 kV detection: 210 + 40 nm	-	naringin, neohesperidin	[41]
		capillary dimension: 50 cm (40 cm effective length) × 75 μm BGS: 25 mM sodium cholate in 150 mM Tris and 15% DMF (pH 7.5) injection: 0.5 psi for 5 s capillary temperature: 25 °C separation voltage: 15 kV detection: 428 nm	-	verteporfin	[42]
		capillary dimension: 37 cm (30 cm effective length) × 50 μm BGS: 25 mM sodium cholate in 300 mM borate and 10% ACN (pH 9.2) injection: +10 kV for 2 s capillary temperature: 20 °C separation voltage: 20 kV detection: 488 nm (λ_ex_)/694 nm (λ_em_)	*linear range*: 0.05–50 mg/L *LOD*: 3.09–4.88 µM	benzoporphyrin derivative mono- and diacids	[43]
		capillary dimension: 60 cm (52.5 cm effective length) × 50 μm BGS: 70 mM sodium cholate in 30 mM sodium tetraborate buffer (pH 9.2) with 25% (*v*/*v*) MeOH or 5.0% (*v*/*v*) n-BuOH injection: 5 kPa for 1 s capillary temperature: 25 °C separation voltage: 25 kV detection: 214 nm	-	palonosetron hydrochloride	[44]
		capillary dimension: 60 cm (50 cm effective length) × 50 μm BGS: 30 mM sodium cholate in 30 mM sodium tetraborate buffer (pH 9.2) with 14% MeOH, 14% EtOH, 9% *n*-PrOH, 11% i-PrOH, 10% *t*-BuOH, 11% acetone, 11% ACN, 6.5% DMF, or 15% DMSO injection: 6 kPa for 1 s capillary temperature: 25 °C separation voltage: 25 kV detection: 214 or 254 nm	*linear range*: 5–50 µg/mL *LOD*: 0.3 µg/mL *LOQ*: 1.0 µg/mL	palonosetron hydrochloride	[45]
		capillary dimension: 60 cm (50 cm effective length) × 50 μm BGS: 30 mM sodium cholate with 1 mM SDS in 30 mM sodium tetraborate (pH 9.2) injection: 10 kPa for 1 or 5 s capillary temperature: 20 °C separation voltage: 25 kV detection: 214 nm	*linear range*: 0.5–50 µg/mL *LOD*: 0.08–0.09 µg/mL *LOQ*: 0.28–0.31 µg/mL	palonosetron hydrochloride	[46]
		capillary dimension: 60 cm (50 cm effective length) × 50 μm BGS: 50 mM sodium cholate and 100 mM SDS in MeOH/ACN (4:1 *v*/*v*) sample injection: 0.5 psi for 5 s capillary temperature: 25 °C separation voltage: 30 kV detection: 200 nm	-	pyraclofos	[47]
		capillary dimension: 30–50 cm (19.5–39.5 cm effective length) × 50 μm BGS: 60 mM sodium cholate in 20 mM CAPS (pH 9.0) and 20% ACN injection: 0.5 psi for 5–90 s separation voltage: 25 kV detection: 220 nm	*SEF*: ~18	Binaphthyl enantiomers, DL-trp	[52]
		capillary dimension: 50 cm (40 cm effective length) × 75 μm BGS: 25 mM sodium cholate in 150 mM Tris (pH 7.5) and 15% (*v*/*v*) DMF sample injection: 0.8 psi vs. −10.3 kV for 120 s capillary temperature: 25 °C separation voltage: 20 kV detection: 428 nm	*LOD*: 10.3 µg/L	verteporfin	[53]
Sodium Taurocholate		capillary dimension: 72 cm effective length × 50 μm BGS: 5 mM sodium taurocholate in 50 mM acetate buffer (pH 2.5) capillary temperature: 30 °C separation voltage: 20 kV detection: 220 nm	-	metyrosine	[48]
		capillary dimension: 72 cm (50 cm effective length) × 50 μm BGS: 5 mM sodium taurocholate in 50 mM sodium dihydrogen phosphate (pH 2.5) capillary temperature: 30 °C separation voltage: 15 kV detection: 220 nm	*linear range*: 50–500 ng/mL *LOD*: 25 ng/mL *LOQ*: 50 ng/mL	arotinolol	[49]
Taurochenodeoxycholic Acid or Glycochenodeoxycholic Acid		capillary dimension: 63 cm (49 cm effective length) × 50 μm BGS: 35 mM taurochenodeoxycholic or glycochenodeoxycholic acid in 0.005 mM phosphate/borate buffer with 10% acetone (pH 9) injection: 5 kV for 7 s separation voltage: 20 kV detection: 232 nm	-	Iron (II) triaza aromatic ligand	[50]
		capillary dimension: 43 cm (29 cm effective length) × 50 μm BGS: 20 mM sodium taurochenodeoxycholate or glycochenodeoxycholate in 0.005 mM phosphate/borate buffer with 10% (*v*/*v*) acetone (pH 3–9) injection: 5 kV for 7 s separation voltage: 15–20 kV detection: 232 nm	-	bis(8-((pyridine-2-methylene)amino)quinoline) iron(II) hexafluorophosphate	[51]
*With Another Chiral Selector*					
Sodium Cholate	γ-CD	capillary dimension: 60 cm (50 cm effective length) × 50 μm BGS: 50–75 mM sodium cholate and 10–20 mM γ-CD in MeOH/H_2_O/ACN (5:4:1 *v*/*v*) sample injection: 0.5 psi for 5 s capillary temperature: 25 °C separation voltage: 30 kV detection: 200 nm	-	profenofos, prothiofos, sulprofos	[47]
	sulfated β-CD and hydroxypropyl β-CD	capillary dimension: 60 cm (53 cm effective length) × 50 μm BGS: 50 mM sodium cholate with 15 mM sulfated β-CD and 5 mM hydroxypropyl β-CD in 20 mM sodium borate (pH 9.0) sample injection: 18 s at a height of 10 cm capillary temperature: 10 °C separation voltage: 30 kV detection: 214 nm	*linear range*: 0.7–400 µg/mL *LOD:* 0.2 µg/mL *LOQ*: 0.7 µg/mL	sertraline	[54]
	heptakis (2,3,6-tri-O-methyl)- β-CD	capillary temperature: 58.5 cm (50 cm effective length) × 50 μm BGS: 100 mM sodium cholate with 20 mM heptakis (2,3,6-tri-*O*-methyl)- β-CD in 100 mM borate (pH 8) and 2 M urea injection: 50 mbar for 2 s capillary temperature: 15 °C separation voltage: 30 kV detection: 210 nm	*linear range*: 10–150 mg/L *LOD*: 3.9 (E2), 4.8 (E1) *LOQ*: 11.8 (E2), 11.8 (E1)	*cis*-bifentrhin	[55]
	human serum albumin	capillary temperature: 60.2 cm (50 cm effective length) × 50 μm BGS: (asp) 12 mM sodium cholate with 0.5% (*v*/*v*) HSA in 12 mM sodium (pH 8.9) and 10% (*v*/*v*) MeOH (glu) 12 mM sodium cholate with 1.6% (*v*/*v*) HSA in 12 mM sodium (pH 9.1) and 5% (*v*/*v*) MeOH capillary temperature: 25 °C separation voltage: −25 kV detection: 488 nm (λ_ex_)/520 nm (λ_em_)	*linear range:* 0.60–160 ng/mL *LOD:* 0.022–0.038 ng/mL *LOQ:* 0.60−1.20 ng/mL	DL-asp, DL-glu	[56]
	34-mer single stranded DNA aptamer	capillary dimension: 60 cm (50 cm effective length) × 75 μm BGS: 50 mM sodium cholate with 10 mM NaCl, 1 mM MgCl_2_, 2.5 mM KCl, 2 mM KH_2_PO_4_ and 10 mM Na_2_HPO_4_ (pH 7.4) injection: 0.5 psi for 6 s capillary temperature: 25 °C separation voltage: 20 kV detection: 280 nm	*linear range*: 0.0625–2 mM *LOD*: 0.0125 mM (D), 0.0153 mM (L)	DL-trp	[57]
Sodium Taurocholate	β-CD	capillary dimension: 57 cm (50 cm effective length) × 50 μm BGS: 30 mM sodium taurocholate with 20 mM β-CD in 80 mM sodium borate buffer (pH 9.3) injection: 0.5 psi for 1 s capillary temperature: 20 °C separation voltage: 20 kV detection: 488 nm (λ_ex_) / 520 nm (λ_em_)	-	20 FITC-derivatized amino acids	[58]
		capillary dimesnion: 67 cm (50 cm effective length) × 50 μm BGS: 40 mM sodium taurocholate with 30 mM β-CD in 40 mM borax (pH 9) and 15% (*v*/*v*) *i*-PrOH injection: 10 kV for 10 s capillary temperature: 22 °C separation voltage: 20 kV detection: 214 nm	*-*	*o*-phthaldiadehyde derivatized amino acids	[59]
		BGS: 12 mM sodium taurocholate with 8 mM β-CD in 5 mM borate (pH 9.2)	*-*	DL-asp, DL-leu	[60]
		BGS: 6 mM sodium taurocholate with 18 mM β-CD in 50 mM sodium borate buffer and 20% (*v*/*v*) MeOH (pH 9.3)	-	DL-asp, DL-ser	[61]
		capillary temperature: 60 cm (50 cm effective length) × 25 μm BGS: 18 mM sodium taurocholate with 12 mM β-CD in 80 mM sodium borate (pH 9.3) injection: 0.3 psi for 2 s capillary temperature: 25 °C separation voltage: 25 kV detection: 520 nm (LIF)	*linear range*: 0.8–50 pM (DL-ala, DL-asp, DL-leu); 1.2–50 (DL-glu) *LOD*: 30 pM (DL-ala, DL-leu); 60 pM (DL-asp, DL-glu) *SEF*: 330–400	DL-ala, DL-asp, DL-glu, DL-leu	[62]
	γ-CD	capillary dimension: 30–60 cm (20–50 cm effective length) × 50 μm BGS: 30 mM sodium taurocholate with 30 mM γ-CD in 80 mM sodium tetraborate (pH 9.2) with 5% ACN injection: 0.5 psi for 4 s capillary temperature: 20 °C separation voltage: 0.5 kV/cm detection: 488 nm (LIF)	*LOD*: 5–25 nM	DL-his, DL-leu, DL-val, Dl-ser, DL-ala, DL-glu, DL-asp	[63]
Sodium Deoxycholate	β-CD	capillary dimension: 50 cm effective length × 50 μm BGS: 60 mM sodium deoxycholate with 30 mM β-CD in 100 mM borate (pH 9.5) injection: at a height differential of 20 cm for 10 s separation voltage: 15 kV detection: 457.9 nm (LIF)	*LOD*: 0.3 nM	D-ser	[64]
	β-CD	capillary dimension: 40 cm effective length × 25 μm BGS: 30 mM sodium deoxycholate with 20 mM β-CD in 35 mM phosphate buffered saline (pH 7.85) and 20% (*v*/*v*) acetonitrile injection: 20 kV for 4 s separation voltage: 20 kV detection: 1.3 V (vs. AgCl/Ag)	-	(RS)-1-phenyl tetrahydrodgen isoquinoline	[65]
	hydroxypropyl-β-CD	capillary dimension: 57 cm (50 cm effective length) × 50 μm BGS: 30 mM sodium deoxycholate with 20 mg/mL HP-β-CD in 35 mM borate (pH 11.5) capillary temperature: 20 °C separation voltage: 25 kV detection: 210 nm	-	sertraline	[66]
	acetyl-β-CD	capillary dimension: 58.5 cm (50 cm effective length) × 50 μm BGS: 75 mM sodium deoxycholate with 15 mM acetyl-β-CD in 100 mM borate (pH 8) and 2 mM urea injection: 50 mbar for 2 s capillary temperature: 25 °C separation voltage: 30 kV detection: 220 nm	*linear range: (external calibration)* 0.5–150 mg/L; *(standard addition)* 0–110 mg/L *LOD:* (R) 0.2 mg/L; (S) 0.3 mg/L *LOQ:* (R) 0.7 mg/L; (S) 0.9 mg/L	bioallenthrin	[67]
	2-hydroxypropyl- β-CD	capillary dimension: 50 cm (40 cm effective length) × 50 μm BGS: 15–25 mM sodium deoxycholate with 40–50 mM HP-β-CD in 100 mM Tris-H_3_PO_4_ (pH 8–9.2) injection: 0.5 psi for 5 s capillary temperature: 25 °C separation voltage: 20 kV detection: 200 nm	-	derivatized dipeptides (ala-gln, tyr-leu, tyr-phe)	[68]
	sulfated β-CD	capillary dimension: 42 cm (36 cm effective length) × 50 μm BGS: 20 mM sodium deoxycholate with 2% (*w*/*v*) sulfated β-CD in 20 mM sodium borate (pH 10) injection: 0.8 psi for 5 s capillary temperature: 20 °C separation voltage: 18 kV detection: 210 nm	*LOD*: (R/S-praziquantel) 50 ng/mL (R/S-trans-4-hydroxypraziquantel) 62.5 ng/mL	praziquantel, *trans*-4-hydroxy-praziquantel	[69]
Sodium Taurodeoxycholate	β-CD	capillary temperature: 50 cm (40 cm effective length) × 75 μm conditioning buffer: 35 mM sodium taurodeoxycholate and 35 mM β-CD in 1.5 M Tris-borate (pH 8.5) with 12.5% (*v*/*v*) IPA BGS: 35 mM sodium taurodeoxycholate with 35 mM β-CD and 0.5% *w*/*v* PEO in 150 mM Tris-borate (pH 8.5) with 12.5% (*v*/*v*) i-PrOH injection: 240 s at 20 cm height separation voltage: 15–18 kV detection: 260 nm	*SEF*: 106–219	FMOC -derivatized amino acids	[70]
	β-CD	capillary temperature: 60 cm (50 cm effective length) × 75 μm BGS: 35 mM sodium taurodeoxycholate with 35 mM β-CD in 150 mM borate (pH 8.5) with 18% (*v*/*v*) *i*-PrOH injection: 0.3 psi for 2 s capillary temperature: 25 °C separation voltage: 25 kV detection: 214 nm	-	FMOC -derivatized amino acids	[71]
*With an Achiral Surfactant and a CS*					
Sodium Taurodeoxycholate	SDS and γ-CD	capillary dimension: 65 cm (50 cm effective length) × 50 μm BGS: 235 nm/0.05 M sodium cholate, 0.05 M SDS and 0.05 γ-CD in 0.1 M CHES buffer (pH 9) and 2 M urea injection: 20 mbar for 1.2 s separation voltage: 15 kV detection: 235 nm	-	chiral polychlorinated biphenyls	[72]
Taurodeoxycholic acid	SDS and β-CD	capillary dimension: 67 cm (50 cm effective length) × 50 μm BGS: 50 mM taurodeoxycholic acid, 50 mM SDS and 30 mM β-CD in 30 mM phosphate buffer/10 mM boric acid (pH 7) injection: 70 mbar for 0.6 s separation voltage: 12 kV detection: 230 nm	*LOD*: 0.06 mM	DL-selenomethionine, DL-selenoethionine	[73]

*Abbreviations*: ACN—acetonitrile; BGS—background solution; *n*-BuOH—*n*-butanol; *t*-BuOH—*tert* butyl alcohol, CAPS—*N*-cyclohexyl-3-aminopropanesulfonic acid; CHES—*N*-cyclohexyl-2-aminoethanesulfonic acid; DMF—dimethylformamide; DMSO—dimethyl sulfoxide; EtOH—ethanol; FMOC-9—fluoroenylmethylchloroformate; HSA—human serum albumin; MeOH—methanol; PEO—poly(ethylene oxide); *i*-PrOH—isopropyl alcohol; *n*-PrOH—*n*-propanol; SDS—sodium dodecyl sulfate; *amino acid abbreviations*: ala—alanine; asp—asparatate; glu—glutamate; his—histidine; leu—leucine; phe—phenylalanine; ser—serine; trp—tryptophan; tyr—tyrosine; val—valine.

## Data Availability

No new data were created or analyzed in this study. Data sharing is not applicable to this article.
